# Changes in abdominal muscles’ thickness from rest to pelvic floor isolated contraction in healthy women

**DOI:** 10.1007/s40477-025-01105-9

**Published:** 2026-01-16

**Authors:** Bruna Bohrer Mozzaquatro, Francesca Chaida Sonda, Fábio Juner Lanferdini, Amanda Zanella de Mello, Suzana Mallmann, Andriéli Aparecida Salbego Lançanova, Luciana Laureano Paiva, Marco Aurélio Vaz, José Geraldo Lopes Ramos

**Affiliations:** 1 Graduate Program in Health Sciences: Gynecology and Obstetrics, Universidade Federal do Rio Grande do Sul (UFRGS), Porto Alegre, Brazil; 2 Biomechanics and Kinesiology Research Group, Exercise Research Laboratory, Universidade Federal do Rio Grande do Sul (UFRGS), Porto Alegre, Brazil; 3 Biomechanics Laboratory, Center of Physical Education and Sports, Universidade Federal de Santa Maria (UFSM), Santa Maria, Brazil; 4 Undergraduate Program in Physiotherapy, Universidade Federal do Rio Grande do Sul (UFRGS), Porto Alegre, Brazil; 5 Department of Physiotherapy, Universidade Federal do Rio Grande do Sul (UFRGS), Porto Alegre, Brazil; 6 Department of Gynecology and Obstetrics, Faculty of Medicine, Universidade Federal Do Rio Grande Do Sul, Porto Alegre, RS Brazil; 7 Department of Health Sciences, Physical Education Course, Universidade de Santa Cruz do Sul, Independence Avenue, 2293, Santa Cruz do Sul, RS 96815-900 Brazil

**Keywords:** Ultrasound, Abdominal muscles, Muscles thickness, Pelvic floor, Pelvic floor contraction, Inter-analyzer reliability

## Abstract

**Objectives:**

To compare muscle thickness of rectus abdominis, internal oblique, external oblique, and transversus abdominis between rest and PFM isolated contraction.

**Design:**

Cross-sectional reliability study.

**Methods:**

Muscle thickness from rectus abdominis, internal oblique, external oblique and transversus abdominis was obtained from 27 physically active women (age: 26.41 ± 0.77 years) with ultrasound imaging at rest and during three maximum PFM contractions. Two independent analyzers, blinded to the conditions, analyzed 3 images from each muscle and condition using Image-J 1.42q software. Muscle thickness was measured three times on each image. Differences between conditions (rest and PFM contraction) were analyzed with a Student t-test. To verify the outcome measures’ inter-analyzer reliability, the intraclass correlation coefficient (ICC), standard error of the measurement (SEM), and minimum detectable change (MDC) were calculated.

**Results:**

TrA showed a significant increase in muscle thickness from rest compared to PFM isolated contraction (*p* < 0.001; *t*:−4.137). However, muscle thickness was similar for the two conditions in rectus abdominis (*p* = 0.093; *t*:1.746), internal oblique (*p* = 0.410; *t*:0.838), and external oblique (*p* = 0.847; *t*:0.196). Excellent reliability was found for muscle thickness at rest (*r* = 0.828–1.000; *p* < 0.001) and during PFM contraction (*r* = 0.997–1.000; *p* = 0.001) for rectus abdominis, internal oblique, external oblique and transversus abdominis muscles. SEM and MDC values from all outcomes were low.

**Conclusion:**

PFM isolated contraction generates a significant contraction of the transversus abdominis muscle, which helps controlling intra-abdominal pressure. The excellent inter-analyzer reliability evidence that abdominal muscles’ muscle thickness is reliable when obtained by different analyzers.

## Introduction

Pelvic floor muscles (PFM) have sexual, childbirth, continence and pelvic organs support roles [1]. PFM’s correct functioning also decreases the incidence of chronic pelvic pain [2]. During these tasks, abdominal muscles act in synergy with PFM, thereby increasing intra-abdominal pressure (IAP) [3]. IAP also increases during the contraction of the abdominal wall muscles (e.g., during exercises involving the core), which increases the need for good functional PFM. More specifically, a functional pelvic floor provides a structural base and prevents the descent of the pelvic organs during IAP increase [4].

However, in clinical practice, the deficiency of one muscle group may lead to a deficiency in the other group, with functional and clinical implications. Therefore, abdominal synergic action has been recommended as an important method to activate the PFM thereby promoting continence while resisting increased IAP, which is generated during functional tasks. In addition, there is evidence that it is possible to stimulate PFM action with the contraction of the abdominal muscle group [3, 5, 6].

Pelvic floor muscle training (PFMT) has been proposed as the first line of treatment for urinary incontinence [7]. In addition, PFMT is recommended during and after pregnancy, as well as for other pelvic floor dysfunctions, prolapse, female sexual dysfunction [7]. During physical activity, there is an abrupt increase in IAP in certain exercises [8]. Therefore, PFM should activate fast fibers to counteract the increase in pressure and prevent the downward movement of the bowel and bladder [3]. In continent women, most likely there is an automatic co-contraction during or before IAP increase [9].

Recruitment of trunk and hip muscles may increase PFMT’s efficiency [5, 7], emphasizing the importance of the abovementioned synergic action of the two muscle groups. There is a large body of evidence demonstrating that, during the activation of abdominal muscles, the PFM are also activated [3, 5, 6]. Similarly, variable patterns of abdominal muscle co-activity occur during PFM contraction. PFM activity has been reported with contraction of all abdominal muscles [10], selective activation of transversus abdominis (TrA) [8], or co-activation of TrA and internal oblique (IO) [11]. The variation in these results may be due to differences in contraction intensity (e.g., gentle vs. maximal contraction), the used instruction and the recording methods of measurement (e.g., intramuscular or surface electromyography). In addition, there is the possibility that different abdominal muscles may be recruited differently during PFM contraction.

Real-time ultrasound imaging is a reliable and valid technique recently used by physical therapists to evaluate muscle structure, function and activation patterns. This method allows for real time study of the muscles as they contract [11, 12]. This is especially important to investigate some deep muscles such as the deep TrA muscle [13, 14].

Ultrasound imaging has recently been used for measuring muscle thickness (MT) of the abdominal muscles [11, 12, 15]. Changes in MT of the abdominal muscles from rest to voluntary effort have been measured as an indicator of muscle activity, which may help differentiate these muscles’ contractility and their force contribution [16, 17].

However, the reliability of ultrasound images can be influenced by the researcher's experience in identifying the same anatomical sites, by different levels of manual pressure performed with the transducer, and by individual variables, such as sufficient relaxation of the muscle to be analyzed [18]. The ultrasound imaging reliability for measuring changes in TrA’s MT has been assessed previously. Costa et al. (2009) reported moderate reliability for MT changes, which support using this non-invasive technique to measure TrA’s and IO’s MT and estimating the relative muscle activity when these muscles are voluntarily contracted [11]. In addition, these authors found high intra-rater reliability for TrA’s and IO’s MT at rest and during PFM contraction in healthy women.

However, understanding abdominal muscles’ function during PFM contraction requires that all abdominal muscles are evaluated, and we found no studies evaluating MT of the external oblique (EO) and the rectus abdominis (RA) muscles at rest and during the isolated contraction of the PFM. In addition, ultrasound studies evaluating the abdominal muscles do not clearly describe important methodological aspects regarding the number of evaluators, training of data acquisition maneuvers, experience of imaging analyzers and data analysis procedures. To the best of our knowledge, no study has evaluated the MT of the abdominal muscles at rest and during the isolated contraction of PFM in healthy women with two analyzers for ultrasound images.

Because of the anatomical characteristics and functional importance for clinical and sports contexts in women, it is important to understand the abdominal muscles’ changes in MT, during different contractile conditions, to extrapolate the findings to clinical practice. In addition, it may allow determining strategies to minimize measurement errors in future evaluation protocols [18]. Therefore, the purposes of this study were: (1) to compare MT of the abdominal muscles (RA, IO, EO, TrA) between rest and PFM isolated contraction; and (2) evaluated ultrasound images’ inter-analyzer reliability. Our hypothesis is that all abdominal muscles should present a significant increase in MT from rest to PFM maximal contraction due to their synergic action controlling IAP during PFM action.

## Material and methods

### Experimental design

**Fig. 1:**
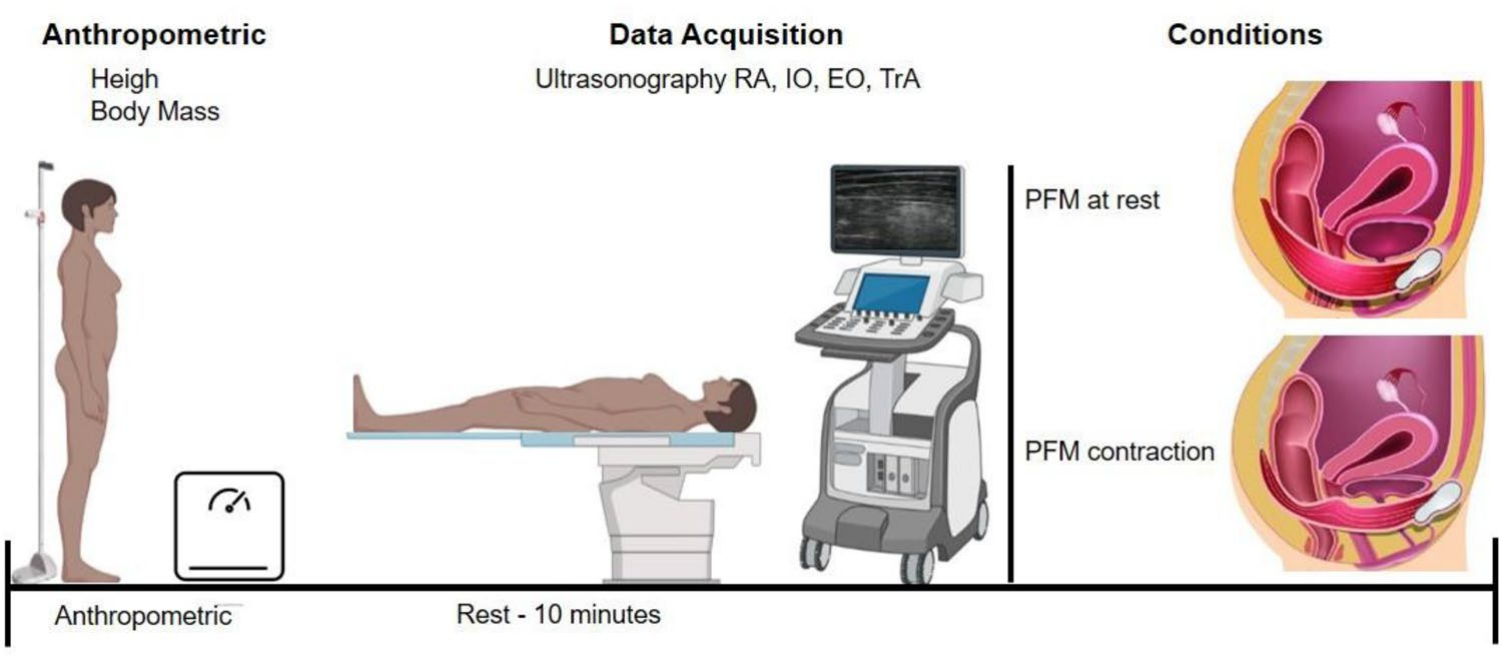
Experimental design of the study’s data collection: (1) Women were relaxed for 10 min; (2) Ultrasound images were collected at rest and during maximum isometric voluntary contraction (MIVC) of pelvic floor muscles (PFM); (3) Three images were collected by the same evaluator

We carried out a cross-sectional study to understand the changes in MT of the abdominal muscles between rest and PFM contraction, and for checking the reliability of our MT measurements. Each female participant came to the laboratory on a single day. Anthropometric data and RA, IO, EO and TrA ultrasound MT were assessed unilaterally by a single evaluator at rest and during PFM maximal isometric voluntary contraction (MIVC) (Fig. 1). This study was conducted according to the Declaration of Helsinki, and all procedures were approved by the local Institutional Research Ethics Committee (project number 2.581.623). All women were informed of the benefits and risks of the investigation before signing an institutionally approved informed consent document to participate in the study.

### Participants

The sample size was based on the study by Lyu et al. [19], who evaluated TrA’s MT at rest and during PFM’s MIVC. Sample size was calculated using G*Power 3.1.3 software (FrauzFaurUniversität Kiel, Germany), and a minimal sample size of 27 subjects was estimated (effect size = 0.25, *α* = 0.05, observed power = 0.80). However, as on a previous study by Da Cuña-Carrera et al. [20] there was a sample loss of 5%, we added 4 more women for eventual sample losses, totaling the sample in 31 volunteers.

All women were healthy university students with no history of chronic diseases or contraindications for performing exercises. Inclusion criteria included age between 18 and 30 years old, being physically active, and not presenting any medical restriction on the performance of maximum tests. Exclusion criteria were pregnancy and having given birth in the previous 12 months, known neurological, neuromuscular or respiratory disorders, low back pain in the previous six months, prolapse of the pelvic organs greater than the second degree, and major surgery of the abdominal or pelvic regions, according to a previous study [21]. In addition, women with sensitive skin, which difficult ultrasound measurements, were excluded. Women were not taking any medication that could influence hormonal and neuromuscular metabolism.

### Data acquisition

**Fig. 2:**
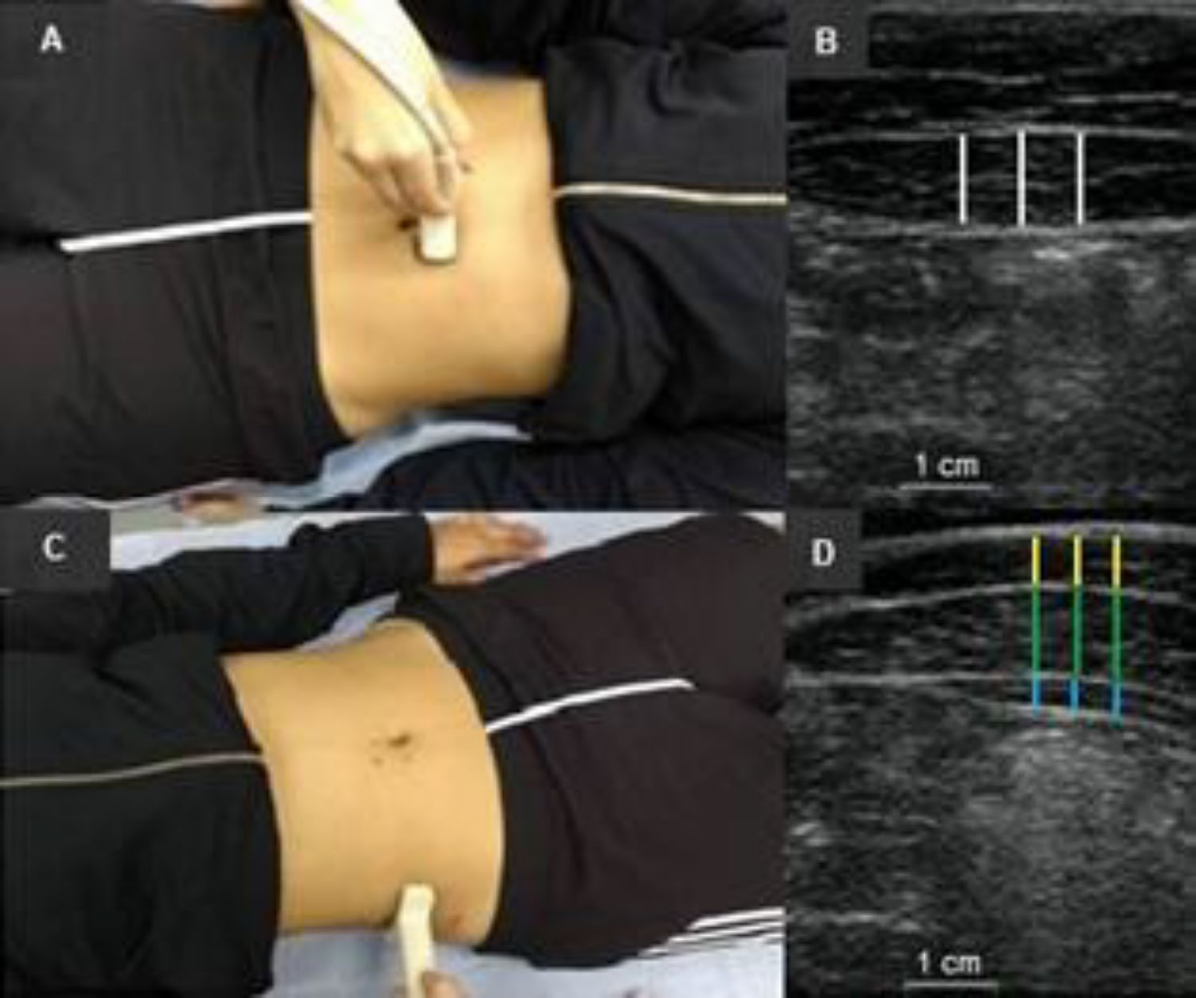
Abdominal muscles’ probe positioning. **A** transducer positioning for rectus abdominis (RA). **B** Muscle thickness (MT) measurement for RA. **C** transducer positioning for internal oblique (IO), external oblique (EO), and transversus abdominis (TrA). **D** MT measurement, from top to bottom, for EO, IO, and TrA

All participants were instructed not to engage in any vigorous physical activity for the previous 48 hours for each evaluation, to avoid the influence of muscle damage on the ultrasonography images [22, 23]. On the data collection day, participants answered a questionnaire containing personal information, anthropometric data, and physical activity level. Before any measurement, subjects rested in the supine position, with the lower limbs extended and relaxed for 15 minutes to allow fluid stabilization. Next, a clinical water-based gel layer was used for acoustic coupling between the ultrasound transducer and the skin above each abdominal muscle [18]. The participants were positioned with one pillow underneath the head and knee and the lumbar spine in neutral position. Great care was taken to determine the specific sites where the images were collected. Anatomical and reference points were marked on the skin (Fig. 2).

The ultrasound imaging measurements were carried out by a physical therapist trained in measuring abdominal MT. The evaluator was not blinded to the volunteers during data collection. An ultrasound system (SSD 4000; ALOKA CO., LTD., Tokyo, Japan) with a linear array probe (60mm; 7.5 MHz) and 90 dB of general gain was used. The frequency, which determines the depth of the sound wave penetration and the image resolution, was kept constant at 8 MHz and a 5 cm depth was determined. Initially, the transducer was positioned 2-3 cm above and to the side of the umbilical scar for RA measurements [13, 20]. Next, for the evaluation of RA’s, IE’s, EO’s and TrA’s MTs, the transducer was positioned transversely between the iliac crest and the 12^th^ rib in the anterior axillary line on the subject’s right side [20].

Ultrasound images were collected at rest and during MIVC of the PFM. Three images were taken from RA, IO, EO and TrA muscles during each condition. The measurements were made on the right side of the abdominal wall at the end of expiration. During the MIVC of the PFM, verbal command (i.e., “squeeze and lift the pelvic floor”) was provided for the subjects. Visual and digital palpation of pelvic floor were performed to guarantee the right execution of the MIVCs [4]. A 2-min rest was observed between MIVCs to avoid muscle fatigue [24].

### Data analysis

Inter-analyzer reliability was obtained from two independent investigators with different experience times in ultrasonography image analysis (Analyzer A = one year and Analyzer B = six months). These analyzers were blinded to the identity of the participants and time-point at which each ultrasonography image was obtained. Each analyzer measured the MT from each muscle once for each of the three images obtained in each time point. Inter-analyzer reliability was obtained from the comparison of the average values of investigator A’s results from each moment of evaluation and the average of investigator B’s results from each evaluation session. A third experienced analyzer re-analyzed the muscle morphology images in case of difficulties/disagreements in the measurements performed between the two independent analyzers.

MT was obtained 3 times from each ultrasonography image from RA, EO, IO and TrA in each condition. Ultrasonography images were analyzed by ImageJ 1.42q software (National Institute of Health, Bethesda, Maryland, USA). MT was defined as the distance between the deep and superficial aponeuroses and was calculated through the mean value of MT obtained from three parallel lines drawn at right angles between the superficial and deep aponeuroses along each ultrasonography image. Mean values were obtained from three ultrasonography images for each muscle to determine MT [20, 22, 25].

### Statistical analysis

The statistical analysis included data mean values (M), standard deviation (SD) values, and coefficient of variation (CV) of all data. The data normality was verified by the Shapiro-Wilk test. All data obtained by the two analyzers were used to verify the inter-analyzer reliability of the outcome measures. Differences between conditions (rest and MIVC of PFM) were analyzed by the paired-sample Student t-test. To verify the outcome measures’ reliability, the intraclass correlation coefficient (ICC 2,1 - Agreement), standard error of the measurement (SEM) and minimum detectable change (MDC) were calculated. The ICC was classified as excellent (r > 0.90); good (r = 0.75 - 0.90); moderate (r = 0.50 - 0.75) or poor (r < 0.50) according to Koo and Li [26]. SEM was estimated using the equation: SEM = SD * √ (1-ICC), according to [27]. The MDC was estimated based on a 95% confidence interval (95%CI), where MDC = 1.96 * SEM [28]. The level of significance adopted for all analyzes was set at 5%. All statistical procedures were performed using the statistical package SPSS 20.0 (IBM, Chicago, USA) for Windows.

## Results

Thirty-one female volunteers met the inclusion criteria and agreed to participate in the study. All participants were undergraduate or graduated students (age: 26.4 ± 0.8 years; body mass: 61.7 ± 6.6 kg; stature: 160 ± 10 cm), physically active (mean of 3 days per week of physical activity), practicing activities such as weight training, running, Pilates, and cross training.

A good to excellent inter-analyzer reliability was observed for most of the outcome variables. IO’s, EO’s and TrA’s MTs at rest presented excellent ICC range values (r=0.993-1.000; *p*<0.001), whereas RA_rest_ (rectus abdominis at rest) inter-analyzer ICC ranged from good to excellent values (r=0.828-1.000; *p*<0.001). Similarly, RA’s, IO’s, EO’s and TrA’s MTs during the MIVC presented excellent ICC range values (r=0.997-1.000; *p*<0.001). In addition, SEM and MDC values were low for all assessed muscles (Table 1).

**Table 1:** Inter-analyzer outcomes of muscle thickness measured by ultrasonography

	Mean ± SD (cm)	ICC (r)	95%CI	*p*-value	SEM (cm)	MDC (cm)	CV (%)
RA_Rest_—Inter-Analyzer	1.05. ± 0.21	0.994	0.988–0.997	< 0.001	0.02	0.03	19.8
IO_Rest_—Inter-Analyzer	0.67 ± 0.11	0.998	0.996–0.999	< 0.001	0.00	0.01	16.5
EO_Rest_—Inter-Analyzer	0.52 ± 0.16	1.000	0.999–1.000	< 0.001	0.00	0.00	29.9
TrA_Rest_—Inter-Analyzer	0.27 ± 0.05	0.998	0.995–0.999	< 0.001	0.00	0.00	19.5
RA_PFMcontraction_—Inter-Analyzer	1.01 ± 0.20	1.000	1.000–1.000	< 0.001	0.00	0.00	19.8
IO_PFMcontraction_—Inter-Analyzer	0.66 ± 0.13	1.000	1.000–1.000	< 0.001	0.00	0.00	19.9
EO_PFMcontraction_—Inter-Analyzer	0.52 ± 0.14	0.997	0.993–0.999	< 0.001	0.01	0.01	26.4
TrA_PFMcontraction_—Inter-Analyzer	0.34 ± 0.10	0.998	0.996–0.999	< 0.001	0.00	0.01	29.2

*RA* rectus abdominis, *EO* external oblique, *IO* internal oblique and *TrA* transversus abdominis, *PFM* pelvic floor muscles, *SD* standard deviation, *ICC* intraclass correlation coefficient, *95%CI* 95% confidence interval, *SEM* standard error of the measurement, *MDC* minimum detectable change, *CV* coefficient of variation

**Fig. 3:**
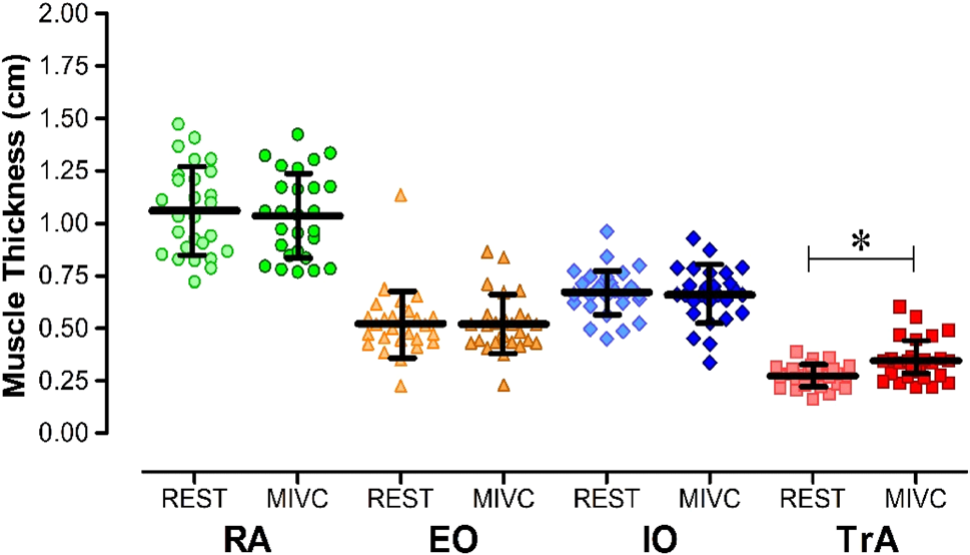
Comparison of muscle thickness from the abdominal muscles at rest and during the pelvic floor muscle (PFM) contraction. * Significant difference between rest and maximal isometric voluntary contraction (MIVC) of PFM; *p* < 0.001

When we compared the MT changes from rest to PFM’s MIVCs, only TrA showed a significant increase in MT (21%; p < 0.001; t: -4.137). The other abdominal muscles (RA, IO and EO) did not show a significant increase in MT from rest to MIVC (*p* = 0.093, t: 1.746; *p* = 0.410, t: 0.838; *p* = 0.847, t: 0.196, respectively), as illustrated in Fig. 3.

## Discussion

This study conducted a comparative analysis of the effects of rest and PFM MIVC on the MT of abdominal muscles, along with evaluating the inter-analyzer reliability of the outcome measures. To the best of our knowledge, our study is the first to evaluate changes in MT (and therefore in contractility) of all abdominal wall muscles during PFM’s MIVC.

Our initial hypothesis was that, during PFM maximal contraction, all abdominal muscles should display a MT increase due to their synergic action with PFM. However, our results demonstrated that TrA was the only abdominal wall muscle that exhibited MT increase during MIVC compared to the resting condition (*p* < 0.001; *t*:−4.137). These results suggest that this muscle might be the most sensitive (and perhaps the most important) from the abdominal wall to control IAP, whereas the other abdominal muscles are probably more prone to act during trunk or core motion [29].

Although previous studies have shown a synergism between PFM and abdominal muscles during PFM contraction [3], most of these studies determined this synergic action by evaluating theses muscles activation by electromyography (EMG). Although EMG is an important clinical tool to evaluate possible neuromuscular problems and is recommended by the International Continence Society [30, 31] ultrasound measures give us a clear idea of the structural changes that occur in the abdominal muscles that are better related with these muscles’ contractility. Hodges et al. [30] study substantiates that ultrasound imaging can discern muscle contraction through observable changes in MT, serving as an indicator of its activity. In other words, ultrasound is a non-invasive, easy to use imaging technique that gives us the possibility of quantifying muscular changes and better determine synergistic actions. In addition, a systematic review that conducted a comparative analysis between the EMG and ultrasound measures for muscle evaluation concluded that ultrasonography is the most sensitive method to evaluate TrA’s muscle function [32]. According to these authors, ultrasound is a real-time, sensitive assessment with good reproducibility, and therefore is a good tool to assess muscle synergistic actions.

Our reasoning for this synergic action was that, when the PFM contract, there should occur an increase in IAP due to the upward displacement of the pelvic organs by the PFM. This upward displacement, therefore, should be responsible for co-activating the abdominal wall muscles to control IAP. However, we did not take into consideration other important structural parameters that also have a direct relation with changes in MT: muscle architecture, which determines its action.

RA is a long strap-like muscle that arises from the pubic crest and inserts superiorly on the xiphoid process and cartilages of the fifth to the seventh ribs [33]. Its fibers are parallel or rectilinear and partially interrupted by three tendinous intersections. Its main action is to flex the trunk during concentric contractions. However, another perhaps important function of this muscle is providing support to all the other abdominal muscles (i.e., IO, EO and TrA) when they contract. These lateral abdominal muscles have their insertions at the midline of the abdomen through a blending of their connective tendinous sheets in the linea alba. As RA is surrounded by two layers (one anterior and one posterior) of these tendinous sheets, it serves as an additional stabilizing point to the linea alba. Nevertheless, changes in RA’s MT should be significant whenever there is a decrease in fascicle length, as during trunk flexion, whereas during PFM’s MIVC, RA’s MT probably should not increase due to its submaximal isometric contraction.

EO and IO are wide and flat muscles constituting the lateral aspect of the abdominal wall. Layered from superficial to deep, EO and IO act during trunk flexion and trunk rotation, due to the oblique direction of their fascicles [33]. Similar to RA, these muscles are more involved in dynamic contractions and their MTs probably increase whenever there is a substantial fiber shortening of these muscles, which is not the case during the PFM’s MIVC.

TrA, however, is the only abdominal muscle that is not related to trunk motion. As described by this muscle’s name, its fibers are located transversally in the abdominal wall. It is the deepest of the lateral abdominal muscles, and its primary function is to increase IAP and stabilize the lumbar region (i.e., providing core stability) [33]. Therefore, the fact that this was the only muscle to show a significant change in MT from rest to PFM’s MIVC is evidence that further supports what has been described in basic kinesiology textbooks regarding TrA function. In other words, TrA was the only to show MT changes because of its primary function of controlling/increasing IAP, which occurs during the PFM’s MIVC.

Assuming that there is a direct relationship between the PFM’s function and/or contractility and the TrA’s contractility (e.g., Bo et al. [4]), and if PFM dysfunction will also reduce TrA function, this may allow evaluating such dysfunction by evaluating these changes in TrA’s MT. In other words, women with PFM contractile problems should show a smaller MT in TrA both at rest and during MIVC compared to healthy women. However, this still needs to be determined.

Our second goal was to determine if MT measures from the abdominal wall muscles were reliable when obtained from two different analyzers. The inter-analyzer reliability analysis displayed good-to-excellent ICC range values, demonstrating that the ultrasound MT measures of the abdominal muscles are reliable when analyzed by different raters. Moreover, low data variability was found in the inter-analyzer results, which were not associated with the second analyst's novice experience (3 months). Nevertheless, less experienced raters may be a complication for MT assessments, especially in populations where muscle quality will be worse than in healthy subjects, such as in elderly and clinical patients, for example, women with urinary incontinence.

Ultrasound emerges as a non-invasive and efficacious technique for real-time assessment of muscle contractility. It notably excels in assessing deep muscles [34], thus proving to be an exceptional method for evaluating the TrA’s structure and function. Furthermore, as previously mentioned, ultrasound offers an indirect means of evaluating muscle activity [30].

### Limitations

The study's primary limitation is that these results are applicable only to healthy women. Therefore, it is not possible to extrapolate our findings to other populations with PFM dysfunctions. Furthermore, we did not control for the effects of the various phases of the menstrual cycle or the use of hormonal contraceptives, which may have an effect on muscle and tendinous tissues functionality. Another limitation is that we were unable to assess the reliability of this measurement among different evaluators during data collection. For future studies, it is recommended that abdominal MT be measured by various evaluators with differing levels of experience in using ultrasound.

### Clinical applications

The outcomes of this study significantly enhance our comprehension of the interplay between abdominal muscles and the PFM. This knowledge holds the potential to enhance the efficacy of treating PFM dysfunctions and offers a valuable resource for rehabilitating women who initially struggle to execute isolated contractions of the PFM. The simultaneous activation of the TrA could increase PFM contraction. It is important to highlight the need for further investigations that explore the reinforcement of TrA as a complementary approach for strengthening the PFM.

## Conclusion

The findings of this study reveal a significant difference in MT of the TrA between the resting state and the MIVC of the PFM. The lack of MT increase of the other abdominal muscles (RA, EO and IO) during PFM’s MIVC suggests that their function is more related to trunk motion, not with a significant co-contraction during PFM’s MIVC. The outcomes emphasize strong levels of both intra-rater and inter-analyzer reliability regarding the measurement of MT for RA, IO, EO, and TrA muscles.

## Data Availability

The datasets generated and/or analyzed during the current study are available from the corresponding author upon reasonable request.
